# Praseodymium(III) sulfate hydroxide, Pr(SO_4_)(OH)

**DOI:** 10.1107/S1600536811000298

**Published:** 2011-01-15

**Authors:** Xiao-Juan Wang, Jian-Wen Cheng

**Affiliations:** a Zhejiang Key Laboratory for Reactive Chemistry on Solid Surfaces, Institute of Physical Chemistry, Zhejiang Normal University, Jinhua, Zhejiang 321004, People’s Republic of China

## Abstract

The title compound, Pr(SO_4_)(OH), obtained under hydro­thermal conditions, consists of Pr^III^ ions coordinated by nine O atoms from six sulfate groups and three hydroxide anions. The bridging mode of the O atoms results in the formation of a three-dimensional framework, stabilized by two O—H⋯O hydrogen-bonding inter­actions.

## Related literature

Lanthanide sulfate hydroxides exhibit a variety of architectures, see: Xu *et al.* (2007 ▶); Zhang *et al.* (2004 ▶). For related structures, see: Yang *et al.* (2005 ▶); Ding *et al.* (2006 ▶); Zhang *et al.* (2004 ▶); Zhang & Lu (2008 ▶).

## Experimental

### 

#### Crystal data

Pr(SO_4_)(OH)
*M* *_r_* = 253.98
Monoclinic, 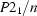

*a* = 4.4891 (18) Å
*b* = 12.484 (5) Å
*c* = 6.894 (3) Å
β = 106.310 (7)°
*V* = 370.8 (3) Å^3^
*Z* = 4
Mo *K*α radiation
μ = 13.59 mm^−1^
*T* = 293 K
0.20 × 0.10 × 0.10 mm

#### Data collection

Bruker APEXII CCD diffractometer
Absorption correction: multi-scan (*SADABS*; Sheldrick, 1996 ▶) *T*_min_ = 0.213, *T*_max_ = 0.257
2849 measured reflections
840 independent reflections
813 reflections with *I* > 2σ(*I*)
*R*_int_ = 0.053

#### Refinement

*R*[*F* ^2^ > 2σ(*F* ^2^)] = 0.033
*wR*(*F* ^2^) = 0.080
*S* = 1.09
840 reflections
65 parameters
H-atom parameters constrained
Δρ_max_ = 1.73 e Å^−3^
Δρ_min_ = −2.15 e Å^−3^

### 

Data collection: *APEX2* (Bruker, 2006 ▶); cell refinement: *SAINT* (Bruker, 2006 ▶); data reduction: *SAINT*; program(s) used to solve structure: *SHELXS97* (Sheldrick, 2008 ▶); program(s) used to refine structure: *SHELXL97* (Sheldrick, 2008 ▶); molecular graphics: *SHELXTL* (Sheldrick, 2008 ▶); software used to prepare material for publication: *SHELXTL*.

## Supplementary Material

Crystal structure: contains datablocks I, global. DOI: 10.1107/S1600536811000298/mg2113sup1.cif

Structure factors: contains datablocks I. DOI: 10.1107/S1600536811000298/mg2113Isup2.hkl

Additional supplementary materials:  crystallographic information; 3D view; checkCIF report

## Figures and Tables

**Table 1:** Hydrogen-bond geometry (Å, °)

*D*—H⋯*A*	*D*—H	H⋯*A*	*D*⋯*A*	*D*—H⋯*A*
O5—H5*A*⋯O3^i^	0.85	2.20	2.630 (7)	111
O5—H5*A*⋯O2^ii^	0.85	2.31	3.082 (7)	152

Symmetry codes: (i) 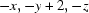; (ii) 
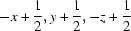
.

**Fig. 1.**
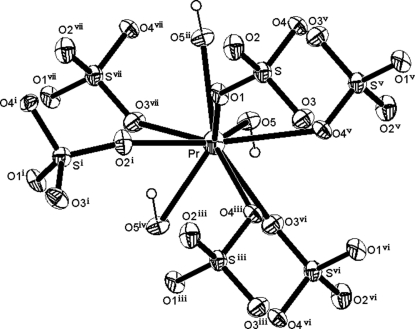
Coordination of Pr in Pr(SO4)(OH). Displacement ellipsoids are drawn at the 30% probability level. [Symmetry codes: (i) x-1/2, -y+3/2, z+1/2; (ii) -x+1, -y+2, -z+1; (iii) x-1, y, z; (iv) -x, -y+2, -z+1; (v) -x+1, -y+2, -z; (vi) -x, -y+2, -z; (vii) x, y, z+1.]

**Fig. 2.**
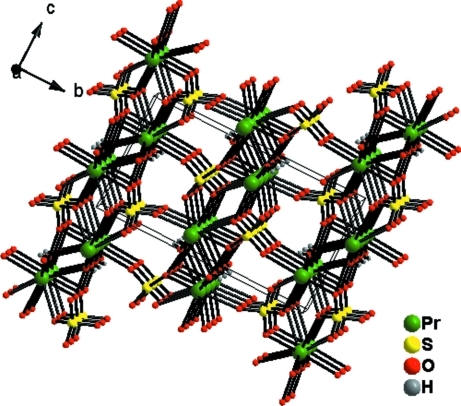
Three-dimensional network in Pr(SO4)(OH).

## supplementary crystallographic information

### Comment

Lanthanide sulfate hydroxides exhibit a variety of architectures (Xu *et al.*, 2007; Zhang *et al.*, 2004).  We report here the compound Pr(SO_4_)(OH), which is isostructural to *Ln*(SO_4_)(OH) (*Ln* = La, Ce, Eu, Nd) (Zhang *et al.*, 2004; Yang *et al.*, 2005;  Ding *et al.*, 2006; Zhang *et al.*, 2008).  The Pr^III^ ion is coordinated in a distorted tricapped trigonal prismatic geometry by the oxygen atoms from six sulfate groups and three hydroxide anions (Fig. 1). All oxygen atoms of the sulfate groups take part in the coordination. The S atom makes four S–O–La linkages through two 2-coordinated oxygen atoms [S–O–La] and two 3-coordinated oxygen atoms [S–(*µ*_3_-O)–La_2_]. The oxygen atoms of the hydroxide groups are four-coordinate, [HO—*µ*_3_-La_3_], linking three different Pr ions. The bridging mode of the oxygen atoms results in a three-dimensional framework, with the H atom of hydroxide anions forming weak O—H···O hydrogen bonds with two O atoms of sulfate groups (Fig. 2).

### Experimental

A mixture of Pr(NO_3_)_3_.6H_2_O (0.25 mmol, 0.1088 g), MnSO_4_.H_2_O (0.2 mmol, 0.0338 g), and H_2_O (15 mL) was sealed in a 25-mL Teflon-lined stainless steel reactor and heated at 443 K for 72 h, and then cooled to room temperature over 3 days. Light-green prismatic crystals were obtained (yield: 32% based on Pr(NO_3_)_3_.6H_2_O).

### Refinement

The oxygen-bound H-atoms were located in the difference Fourier map and refined with the O—H distance restrained to 0.85 Å [*U*_iso_(H) = 1.2*U*_eq_(O)].

### Crystal data

**Table:**

Pr(SO_4_)(OH)	*F*(000) = 464
*M**_r_* = 253.98	*D*_x_ = 4.550 Mg m^−^^3^
Monoclinic, *P*2_1_/*n*	Mo *K*α radiation, λ = 0.71073 Å
Hall symbol: -P 2yn	Cell parameters from 1066 reflections
*a* = 4.4891 (18) Å	θ = 3.3–27.4°
*b* = 12.484 (5) Å	µ = 13.59 mm^−^^1^
*c* = 6.894 (3) Å	*T* = 293 K
β = 106.310 (7)°	Prism, light green
*V* = 370.8 (3) Å^3^	0.20 × 0.10 × 0.10 mm
*Z* = 4	

### Data collection

**Table:**

Bruker APEXII CCD diffractometer	840 independent reflections
Radiation source: fine-focus sealed tube	813 reflections with *I* > 2σ(*I*)
graphite	*R*_int_ = 0.053
ω scans	θ_max_ = 27.4°, θ_min_ = 3.3°
Absorption correction: multi-scan (*SADABS*; Sheldrick, 1996)	*h* = −5→5
*T*_min_ = 0.213, *T*_max_ = 0.257	*k* = −14→16
2849 measured reflections	*l* = −8→8

### Refinement

**Table:**

Refinement on *F*^2^	Secondary atom site location: difference Fourier map
Least-squares matrix: full	Hydrogen site location: inferred from neighbouring sites
*R*[*F*^2^ > 2σ(*F*^2^)] = 0.033	H-atom parameters constrained
*wR*(*F*^2^) = 0.080	*w* = 1/[σ^2^(*F*_o_^2^) + (0.0348*P*)^2^ + 1.2539*P*] where *P* = (*F*_o_^2^ + 2*F*_c_^2^)/3
*S* = 1.09	(Δ/σ)_max_ = 0.001
840 reflections	Δρ_max_ = 1.73 e Å^−^^3^
65 parameters	Δρ_min_ = −2.15 e Å^−^^3^
0 restraints	Extinction correction: *SHELXL97* (Sheldrick, 2008), Fc^*^=kFc[1+0.001xFc^2^λ^3^/sin(2θ)]^-1/4^
Primary atom site location: structure-invariant direct methods	Extinction coefficient: 0.0303 (17)

### Special details

**Table:**

Geometry. All e.s.d.'s (except the e.s.d. in the dihedral angle between two l.s. planes) are estimated using the full covariance matrix. The cell e.s.d.'s are taken into account individually in the estimation of e.s.d.'s in distances, angles and torsion angles; correlations between e.s.d.'s in cell parameters are only used when they are defined by crystal symmetry. An approximate (isotropic) treatment of cell e.s.d.'s is used for estimating e.s.d.'s involving l.s. planes.
Refinement. Refinement of *F*^2^ against ALL reflections. The weighted *R*-factor *wR* and goodness of fit *S* are based on *F*^2^, conventional *R*-factors *R* are based on *F*, with *F* set to zero for negative *F*^2^. The threshold expression of *F*^2^ > σ(*F*^2^) is used only for calculating *R*-factors(gt) *etc*. and is not relevant to the choice of reflections for refinement. *R*-factors based on *F*^2^ are statistically about twice as large as those based on *F*, and *R*- factors based on ALL data will be even larger.

### Fractional atomic coordinates and isotropic or equivalent isotropic displacement parameters (Å^2^)

**Table:**

	*x*	*y*	*z*	*U*_iso_*/*U*_eq_	
Pr	0.14215 (8)	0.93511 (2)	0.30120 (5)	0.0103 (2)	
S	0.4863 (3)	0.85448 (11)	−0.1115 (2)	0.0100 (3)	
O1	0.3690 (11)	0.8345 (4)	0.0615 (6)	0.0156 (9)	
O2	0.5908 (12)	0.7551 (4)	−0.1798 (7)	0.0189 (10)	
O3	0.2507 (11)	0.9045 (4)	−0.2785 (7)	0.0179 (10)	
O4	0.7557 (12)	0.9298 (3)	−0.0482 (8)	0.0137 (10)	
O5	0.3035 (11)	1.0859 (4)	0.5390 (7)	0.0141 (9)	
H5A	0.1434	1.1193	0.5488	0.021*	

### Atomic displacement parameters (Å^2^)

**Table:**

	*U*^11^	*U*^22^	*U*^33^	*U*^12^	*U*^13^	*U*^23^
Pr	0.0129 (3)	0.0104 (3)	0.0083 (3)	0.00025 (10)	0.00418 (19)	−0.00100 (9)
S	0.0118 (7)	0.0098 (7)	0.0091 (7)	−0.0002 (5)	0.0041 (6)	0.0002 (5)
O1	0.020 (2)	0.020 (2)	0.012 (2)	0.002 (2)	0.0142 (19)	0.0010 (17)
O2	0.027 (2)	0.014 (2)	0.020 (2)	0.001 (2)	0.011 (2)	−0.0016 (17)
O3	0.017 (2)	0.023 (2)	0.015 (2)	0.005 (2)	0.0060 (19)	0.0030 (19)
O4	0.013 (2)	0.013 (2)	0.016 (2)	−0.0039 (15)	0.005 (2)	−0.0022 (14)
O5	0.014 (2)	0.017 (2)	0.012 (2)	0.0037 (18)	0.0044 (18)	−0.0042 (17)

### Geometric parameters (Å, °)

**Table:**

Pr—O2^i^	2.393 (5)	S—O1	1.455 (5)
Pr—O5^ii^	2.436 (5)	S—O3	1.466 (5)
Pr—O5	2.468 (5)	S—O4	1.497 (5)
Pr—O1	2.508 (4)	O2—Pr^viii^	2.393 (5)
Pr—O4^iii^	2.543 (5)	O3—Pr^vi^	2.643 (5)
Pr—O5^iv^	2.554 (5)	O3—Pr^ix^	2.826 (5)
Pr—O4^v^	2.558 (5)	O4—Pr^x^	2.543 (5)
Pr—O3^vi^	2.643 (5)	O4—Pr^v^	2.558 (5)
Pr—O3^vii^	2.826 (5)	O5—Pr^ii^	2.436 (5)
Pr—Pr^iv^	3.7056 (12)	O5—Pr^iv^	2.554 (5)
Pr—Pr^ii^	3.9388 (12)	O5—H5A	0.8500
S—O2	1.450 (5)		
			
O2^i^—Pr—O5^ii^	88.30 (16)	O5—Pr—Pr^iv^	43.35 (12)
O2^i^—Pr—O5	137.33 (16)	O1—Pr—Pr^iv^	173.83 (10)
O5^ii^—Pr—O5	73.13 (18)	O4^iii^—Pr—Pr^iv^	115.28 (12)
O2^i^—Pr—O1	66.58 (17)	O5^iv^—Pr—Pr^iv^	41.55 (10)
O5^ii^—Pr—O1	72.04 (15)	O4^v^—Pr—Pr^iv^	112.38 (11)
O5—Pr—O1	136.21 (16)	O3^vi^—Pr—Pr^iv^	49.45 (10)
O2^i^—Pr—O4^iii^	88.59 (15)	O3^vii^—Pr—Pr^iv^	45.31 (10)
O5^ii^—Pr—O4^iii^	139.79 (17)	O2^i^—Pr—Pr^ii^	116.25 (12)
O5—Pr—O4^iii^	130.10 (14)	O5^ii^—Pr—Pr^ii^	36.83 (11)
O1—Pr—O4^iii^	70.01 (15)	O5—Pr—Pr^ii^	36.29 (11)
O2^i^—Pr—O5^iv^	77.01 (17)	O1—Pr—Pr^ii^	105.16 (11)
O5^ii^—Pr—O5^iv^	128.20 (19)	O4^iii^—Pr—Pr^ii^	151.15 (10)
O5—Pr—O5^iv^	84.90 (16)	O5^iv^—Pr—Pr^ii^	109.08 (11)
O1—Pr—O5^iv^	138.17 (15)	O4^v^—Pr—Pr^ii^	86.32 (11)
O4^iii^—Pr—O5^iv^	89.84 (16)	O3^vi^—Pr—Pr^ii^	97.46 (11)
O2^i^—Pr—O4^v^	136.90 (16)	O3^vii^—Pr—Pr^ii^	57.99 (11)
O5^ii^—Pr—O4^v^	91.33 (16)	Pr^iv^—Pr—Pr^ii^	71.85 (3)
O5—Pr—O4^v^	82.80 (16)	O2—S—O1	110.1 (3)
O1—Pr—O4^v^	72.38 (15)	O2—S—O3	109.7 (3)
O4^iii^—Pr—O4^v^	64.96 (17)	O1—S—O3	111.7 (3)
O5^iv^—Pr—O4^v^	132.35 (15)	O2—S—O4	108.9 (3)
O2^i^—Pr—O3^vi^	132.98 (17)	O1—S—O4	108.5 (3)
O5^ii^—Pr—O3^vi^	133.47 (15)	O3—S—O4	107.9 (3)
O5—Pr—O3^vi^	61.84 (16)	S—O1—Pr	139.7 (3)
O1—Pr—O3^vi^	136.71 (14)	S—O2—Pr^viii^	155.7 (3)
O4^iii^—Pr—O3^vi^	72.40 (15)	S—O3—Pr^vi^	133.7 (3)
O5^iv^—Pr—O3^vi^	60.84 (15)	S—O3—Pr^ix^	137.8 (3)
O4^v^—Pr—O3^vi^	72.83 (16)	Pr^vi^—O3—Pr^ix^	85.24 (13)
O2^i^—Pr—O3^vii^	78.53 (16)	S—O4—Pr^x^	125.0 (3)
O5^ii^—Pr—O3^vii^	70.23 (15)	S—O4—Pr^v^	120.0 (3)
O5—Pr—O3^vii^	59.19 (16)	Pr^x^—O4—Pr^v^	115.04 (17)
O1—Pr—O3^vii^	128.53 (14)	Pr^ii^—O5—Pr	106.87 (18)
O4^iii^—Pr—O3^vii^	147.45 (16)	Pr^ii^—O5—Pr^iv^	128.20 (19)
O5^iv^—Pr—O3^vii^	58.29 (15)	Pr—O5—Pr^iv^	95.10 (16)
O4^v^—Pr—O3^vii^	140.86 (15)	Pr^ii^—O5—H5A	142.8
O3^vi^—Pr—O3^vii^	94.76 (13)	Pr—O5—H5A	109.3
O2^i^—Pr—Pr^iv^	109.56 (13)	Pr^iv^—O5—H5A	40.0
O5^ii^—Pr—Pr^iv^	103.47 (11)		
			
O2—S—O1—Pr	179.4 (4)	O1—S—O4—Pr^x^	33.7 (4)
O3—S—O1—Pr	−58.5 (5)	O3—S—O4—Pr^x^	154.9 (3)
O4—S—O1—Pr	60.3 (5)	O2—S—O4—Pr^v^	93.2 (3)
O2^i^—Pr—O1—S	167.8 (5)	O1—S—O4—Pr^v^	−147.0 (3)
O5^ii^—Pr—O1—S	−96.1 (4)	O3—S—O4—Pr^v^	−25.8 (4)
O5—Pr—O1—S	−57.3 (5)	O2^i^—Pr—O5—Pr^ii^	68.0 (3)
O4^iii^—Pr—O1—S	70.4 (4)	O5^ii^—Pr—O5—Pr^ii^	0.0
O5^iv^—Pr—O1—S	136.1 (4)	O1—Pr—O5—Pr^ii^	−38.5 (3)
O4^v^—Pr—O1—S	1.3 (4)	O4^iii^—Pr—O5—Pr^ii^	−141.99 (18)
O3^vi^—Pr—O1—S	39.2 (5)	O5^iv^—Pr—O5—Pr^ii^	132.5 (2)
O3^vii^—Pr—O1—S	−140.6 (4)	O4^v^—Pr—O5—Pr^ii^	−93.60 (19)
Pr^iv^—Pr—O1—S	−140.0 (7)	O3^vi^—Pr—O5—Pr^ii^	−167.9 (2)
Pr^ii^—Pr—O1—S	−79.7 (4)	O3^vii^—Pr—O5—Pr^ii^	76.67 (19)
O1—S—O2—Pr^viii^	13.5 (8)	Pr^iv^—Pr—O5—Pr^ii^	132.5 (2)
O3—S—O2—Pr^viii^	−109.8 (7)	O2^i^—Pr—O5—Pr^iv^	−64.5 (3)
O4—S—O2—Pr^viii^	132.3 (7)	O5^ii^—Pr—O5—Pr^iv^	−132.5 (2)
O2—S—O3—Pr^vi^	168.4 (4)	O1—Pr—O5—Pr^iv^	−171.07 (15)
O1—S—O3—Pr^vi^	46.0 (5)	O4^iii^—Pr—O5—Pr^iv^	85.5 (2)
O4—S—O3—Pr^vi^	−73.1 (4)	O5^iv^—Pr—O5—Pr^iv^	0.0
O2—S—O3—Pr^ix^	−39.4 (5)	O4^v^—Pr—O5—Pr^iv^	133.87 (16)
O1—S—O3—Pr^ix^	−161.8 (4)	O3^vi^—Pr—O5—Pr^iv^	59.53 (16)
O4—S—O3—Pr^ix^	79.1 (5)	O3^vii^—Pr—O5—Pr^iv^	−55.86 (15)
O2—S—O4—Pr^x^	−86.1 (4)	Pr^ii^—Pr—O5—Pr^iv^	−132.5 (2)

Symmetry codes: (i) *x*−1/2, −*y*+3/2, *z*+1/2; (ii) −*x*+1, −*y*+2, −*z*+1; (iii) *x*−1, *y*, *z*; (iv) −*x*, −*y*+2, −*z*+1; (v) −*x*+1, −*y*+2, −*z*; (vi) −*x*, −*y*+2, −*z*; (vii) *x*, *y*, *z*+1; (viii) *x*+1/2, −*y*+3/2, *z*−1/2; (ix) *x*, *y*, *z*−1; (x) *x*+1, *y*, *z*.

### Hydrogen-bond geometry (Å, °)

**Table:**

*D*—H···*A*	*D*—H	H···*A*	*D*···*A*	*D*—H···*A*
O5—H5A···O3^vi^	0.85	2.20	2.630 (7)	111
O5—H5A···O2^xi^	0.85	2.31	3.082 (7)	152

Symmetry codes: (vi) −*x*, −*y*+2, −*z*; (xi) −*x*+1/2, *y*+1/2, −*z*+1/2.
